# A Narrative Review of Risk Assessment Approaches for Implantable Defibrillator Therapy to Prevent Sudden Cardiac Death in Acute Myocardial Infarction

**DOI:** 10.1155/crp/1981993

**Published:** 2026-06-14

**Authors:** Nasim Kakavand, Yasaman Shojaei, Mohammadamin Sadri, Sina Rouhani, Razieh Hassannejad, Hamidreza Roohafza, Masoumeh Sadeghi

**Affiliations:** ^1^ Isfahan Cardiovascular Research Center, Cardiovascular Research Institute, Isfahan University of Medical Sciences, Isfahan, Iran, mui.ac.ir; ^2^ Heart Failure Research Center, Cardiovascular Research Institute, Isfahan University of Medical Sciences, Isfahan, Iran, mui.ac.ir; ^3^ Cardiac Rehabilitation Research Center, Cardiovascular Research Institute, Isfahan University of Medical Sciences, Isfahan, Iran, mui.ac.ir; ^4^ Interventional Cardiology Research Center, Cardiovascular Research Institute, Isfahan University of Medical Sciences, Isfahan, Iran, mui.ac.ir

**Keywords:** implantable cardioverter‐defibrillator, ST‐elevation myocardial infarction, sudden cardiac death

## Abstract

Despite considerable progress in managing ST‐elevation myocardial infarction (STEMI), sudden cardiac death (SCD) remains a major clinical challenge. Although implantable cardioverter‐defibrillator (ICD) therapy, guided primarily by left ventricular ejection fraction (LVEF), has been proven to reduce mortality in landmark trials (e.g., MADIT, MUSTT, and MADIT‐II), the modest sensitivity and specificity of LVEF limit its utility as a sole risk stratifier. Emerging clinical trends highlight the progression from traditional LVEF‐based models to more comprehensive risk assessment strategies. This review critically examines additional modalities, including advanced cardiac magnetic resonance imaging, echocardiography, myocardial biomarkers, detailed electrocardiographic parameters, and assessments of myocardial tissue heterogeneity, as well as patient‐specific factors such as age, sex, and prehospital cardiac arrest history. Furthermore, we discuss recent advances, particularly the integration of artificial intelligence and machine learning algorithms, that help to enhance risk prediction and optimize ICD therapy decisions. By combining these novel approaches into a comprehensive risk assessment framework, our review promotes a more personalized strategy to identify STEMI patients most likely to benefit from ICD implantation, improve survival outcomes, and reduce unnecessary interventions. Future research should focus on validating these integrative models in large prospective cohorts and refining current guidelines for ICD therapy post‐STEMI.

## 1. Introduction

Recent decades have witnessed innovative advances in the management of ST‐segment elevation myocardial infarction (STEMI), with rapid revascularization and enhanced pharmacotherapy markedly improving patient survival [1]. Nevertheless, sudden cardiac death (SCD) continues to present a major challenge post‐STEMI [2]. For instance, the PARADISE‐MI trial reported sudden death/resuscitated arrest in 2.6% over 22 months, versus 7.4% in the older VALIANT cohort in post‐myocardial infarction (MI) patients [3].

Traditionally, left ventricular ejection fraction (LVEF) was essential in selecting patients for prophylactic implantable cardioverter‐defibrillator (ICD) therapy [4]. Landmark studies, including MADIT II and SCD‐HeFT, have established guidelines that delay ICD implantation until at least 40 days after MI and 90 days postrevascularization, thereby allowing time for potential recovery of left ventricular function following primary percutaneous coronary intervention (PCI) [5, 6]. However, the reliance on LVEF as the sole risk stratifier is increasingly recognized as inefficient, since most patients who experience SCD have mildly or moderately reduced LVEF [7]. Meta‐analyses report only modest sensitivity (approximately 59%) and specificity (around 78%) of LVEF in predicting arrhythmic events [2, 8], and only a minority of patients with LVEF < 35% eventually receive appropriate ICD therapy [1]. Importantly, the predictive value of LVEF is time‐dependent. Early post‐MI LVEF may recover due to myocardial stunning, whereas long‐term remodeling may increase arrhythmic risk [9, 10]. Moreover, while the risk of SCD peaks within the first 30 days post‐MI, early ICD implantation has not demonstrated a clear survival benefit, likely due to limitations in current risk stratifiers that fail to adequately distinguish between arrhythmic and nonarrhythmic deaths [11].

This narrative review aimed to investigate additional risk stratification modalities, including advanced imaging, biomarker evaluation, and electrophysiological testing, that may complement or even surpass LVEF in predicting SCD risk. By integrating these novel parameters, we propose a more comprehensive and personalized approach to selecting STEMI patients for ICD therapy.

## 2. Methods

This narrative review utilized a PubMed search performed until February 2026 using the following search term:

((“ST‐Elevation Myocardial Infarction” OR “STEMI” OR “Acute Coronary Syndrome”) AND (“Implantable Cardioverter Defibrillator” OR “ICD” OR “Defibrillators, Implantable” OR “Sudden Cardiac Death” OR “SCD”)).

Inclusion criteria prioritized studies evaluating risk stratification modalities beyond LVEF in post‐STEMI patients. Exclusion criteria included case reports, case series, and studies not published in English. Disagreements during article selection were resolved by consensus among the authors.

### 2.1. Trends in Implantable Defibrillators for SCD Prevention

Prevention of SCD after MI remains both challenging and critical. Several clinical trials have proven the efficacy of ICDs for primary or secondary prevention in post‐MI patients. The first large‐scale trial to investigate ICD efficacy for primary prevention was MADIT, which enrolled 196 patients (New York Heart Association [NYHA] Classes I–III) with a history of MI, LVEF ≤ 35%, documented asymptomatic unsustained ventricular tachycardia (VT), and inducible, nonsuppressible ventricular tachyarrhythmia during electrophysiological testing [12]. Patients were randomly assigned to receive either an ICD or conventional medical treatment; after 27 months of follow‐up, the hazard ratio (HR) for overall mortality was 0.46, indicating a clear survival benefit with ICD therapy [12]. Subsequently, the Multicenter Unsustained Tachycardia Trial (MUSTT) demonstrated that electrophysiologically guided antiarrhythmic therapy using ICDs significantly reduced the risk of SCD in high‐risk patients with coronary artery disease (CAD) [13]. In this study, 704 patients with CAD, LVEF ≤ 40%, and asymptomatic nonsustained VT were randomized into different treatment groups, with ICD‐treated patients showing a markedly lower risk of cardiac arrest or arrhythmic death (relative risk: 0.24) [13].

MADIT‐II further examined the survival benefit of ICD implantation in post‐MI patients without mandatory electrophysiological testing [5]. In this trial, 1232 patients with a history of MI and reduced LVEF (< 30%) were randomized to ICD or medical therapy, with a 31% reduction in all‐cause mortality observed in the ICD group after an average follow‐up of 20 months [5]. The Defibrillator in Acute Myocardial Infarction Trial (DINAMIT) investigated early ICD implantation in patients (within 6–40 days post‐MI) with reduced EF (< 35%) and evidence of autonomic dysfunction [14]. Although the primary outcome of all‐cause mortality was similar between the ICD and conventional therapy groups, the prespecified secondary outcome of arrhythmic death was significantly lower in the ICD group [14].

An analysis from MUSTT, involving patients who did not receive pharmacologic antiarrhythmic therapy or an ICD, emphasized the multifactorial nature of mortality in chronic CAD [15]. This study highlighted the importance of historical clinical factors, beyond LVEF alone, for predicting prognosis, noting that patients with EF between 30% and 40% might be at elevated risk when additional factors are present [15]. Additionally, a retrospective analysis of 128 patients experiencing early ventricular arrhythmias (VAs) following acute MI identified clinical factors such as older age, female gender, previous coronary artery bypass grafting (CABG), or MI, and presentation with non‐ST‐elevation MI or VT as being associated with poorer overall survival [16]. Interestingly, while LVEF did not significantly predict mortality (HR = 1; *p* = 0.86), post‐MI ventricular fibrillation (VF) was linked to a more favorable long‐term prognosis compared to VT (HR 0.37, *p* = 0.001), suggesting that arrhythmia type may influence outcomes more than LVEF alone [16].

A retrospective, single‐center study of 77 acute MI patients with LVEF ≤ 35% assessed the implications of early ICD implantation for primary prevention of SCD [17]. The findings revealed that all patients with low ejection fraction (EF) immediately after MI remained in the same category after 40 days, with the majority (96.1%) ultimately requiring an ICD [17]. Early ICD implantation could reduce unnecessary exposure to SCD risk during this vulnerable period, suggesting that revisiting current guidelines to allow for earlier intervention in high‐risk patients may improve outcomes [17].

### 2.2. Current Guidelines for ICD Implantation Post‐MI

Current guidelines recommend the ICD implantation in patients with significant left ventricular (LV) dysfunction that persists 40 days after an acute MI or 3 months after revascularization [18]. The indications for ICD placement after an acute coronary syndrome are as follows.

#### 2.2.1. Primary Prevention

- Patients who have had a previous MI, have an LVEF of less than 35% despite receiving the optimal medical treatment, are classified as NYHA class II or III, and are more than 40 days past their last MI or more than 90 days past their most recent revascularization procedure
- Patients who have had a previous MI, have an LVEF of less than 30% despite receiving the optimal medical treatment, are classified as NYHA class I, and are more than 40 days past their last MI or more than 90 days past their most recent revascularization procedure

#### 2.2.2. Secondary Prevention

- Patients who have survived SCA secondary to VA and do not have a reversible etiology
- Patients with nonsustained VAs (NSVT), a history of MI, LVEF < 40%, and inducible VA during an electrophysiological study

### 2.3. Enhancing Risk Stratification for ICD Implantation Post‐STEMI

Despite notable improvements in STEMI care, SCD remains a persistent concern. The traditional reliance on LVEF as the main criterion is increasingly seen as insufficient. Here, we focus on STEMI patients and explore additional modalities to refine risk stratification. Table 1 summarizes various risk stratification modalities beyond LVEF.

**TABLE 1:** Risk stratification modalities beyond LVEF in STEMI patients.

Domain	Modality/factor	Key findings	Clinical implication	Limitations
Patient‐specific factors	Sex	Women have lower inducible VT (22.1% vs 33.4%) and lower arrhythmic events (1.6% vs 26.5%) compared to men	Suggests need for sex‐specific risk stratification	Underrepresentation of women in studies
	Age	Older age ⟶ higher nonarrhythmic mortality; reduced proportional SCD risk	May reduce net benefit of ICD in elderly	Competing risks complicate decision‐making
	Prehospital cardiac arrest	Higher mortality (13.6% vs 8.7%) post‐STEMI	Identifies high‐risk subgroup needing closer monitoring	Limited integration into current models

Noninvasive imaging	Echocardiography (GLS, MD)	GLS and mechanical dispersion predict arrhythmias independent of LVEF	Widely available, cost‐effective risk markers	Operator variability; limited fibrosis detection
	Speckle‐Tracking Echo	Border zone strain impairment predicts ICD therapy/cardiac death	Detects regional heterogeneity	Small study sizes
	CMR (LGE)	LGE extent strongly predicts SCD (↑ risk per 5% increment)	Gold standard for scar characterization	Limited availability, cost
	SPECT/PET	Scar burden predicts SCD and ICD therapies (independent of EF)	Functional + structural risk assessment	Radiation, access limitations

Biomarkers	hs‐CRP, sST2, BNP, hsTnT, NT‐proBNP	Independently predict SCD even with preserved EF	Add biological risk dimension	Mechanistic role unclear Lacks prospective validation for routine post‐MI use
	Proteomic panel (3 proteins)	C‐statistic 0.752 vs 0.548 for LVEF	Improved risk discrimination, especially preserved EF	Early‐stage validation

Electrocardiographic markers	ECG parameters (HR, QRS, QTc, HRV, TWA, etc.)	Multiple abnormalities predict SCD (HR up to 6.6 for cumulative risk)	Useful screening tools	Limited specificity alone
	NIRFs + PVS strategy	100% sensitivity & NPV for arrhythmic events	May reduce unnecessary ICD implantation	Requires invasive follow‐up

Invasive testing	Electrophysiology study (EPS)	Inducible VT ⟶ high event rate (22% vs 4.3%)	Direct identification of arrhythmogenic substrate	Invasive, not widely used routinely

Artificial intelligence	AI‐ECG models	High accuracy for SCD prediction (AUC 0.93–0.99)	Promising next‐generation risk tools	Requires prospective validation
	ML (hemodynamic + clinical data)	AUC > 0.80 for mortality prediction	Enhances multimodal prediction	Generalizability concerns
	Wearables & CIED‐AI	High AF detection (sens ∼95%, spec ∼97%)	Enables continuous monitoring & early detection	Data integration challenges

*Note:* ECG, electrocardiogram; hsTnT, high‐sensitivity troponin T; NIRFs, noninvasive risk factors; NSVT, nonsustained ventricular tachycardia; QTc, corrected QT interval.

Abbreviations: AF, atrial fibrillation; AI, artificial intelligence; AUC, area under the curve; BNP, B‐type natriuretic peptide; CAD, coronary artery disease; CIED, cardiac implantable electronic device; CMR, cardiac magnetic resonance; CRT, cardiac resynchronization therapy; EF, ejection fraction; GLS, global longitudinal strain; HR, heart rate; HRV, heart rate variability; hs‐CRP, high‐sensitivity C‐reactive protein; ICD, implantable cardioverter‐defibrillator; LGE, late gadolinium enhancement; LV, left ventricle; LVEF, left ventricular ejection fraction; LVH, left ventricular hypertrophy; MD, mechanical dispersion; ML, machine learning; MPI, myocardial perfusion imaging; NT‐proBNP, N‐terminal pro‐brain natriuretic peptide; PET, positron emission tomography; PVS, programmed ventricular stimulation; RCA, resuscitated cardiac arrest; SCD, sudden cardiac death; SPECT, single‐photon emission computed tomography; STEMI, ST‐elevation myocardial infarction; TWA, T‐wave alternans; VF, ventricular fibrillation; VT, ventricular tachycardia.

### 2.4. Role of Patient‐Specific Factors as Predictors

#### 2.4.1. Sex Differences

A prospective study examined the role of electrophysiology studies (EPS) in early SCD prevention among STEMI patients with LVEF ≤ 40% [19]. In this study of 403 patients (16.9% female), EPS identified inducible monomorphic VT, leading to ICD implantation based on positive findings. Women, despite being older, exhibited similar LVEF values compared to men. However, inducible VT was found in 22.1% of women versus 33.4% of men, and appropriate ICD activations occurred more frequently in men (36.6% vs. 5.9%). The adjusted cumulative incidence of the primary endpoint (VT/VF, cardiac arrest, or SCD) was significantly lower in women (1.6%) compared to men (26.5%) [19]. These observations, along with other studies [20–22], highlight the need for sex‐specific risk stratification criteria, possibly reflecting hormonal influences and differences in the myocardial substrate.

#### 2.4.2. Age Considerations

With advancing age, the proportional risk of sudden arrhythmic death decreases relative to competing risks of nonsudden mortality, potentially diminishing the absolute benefit of ICD implantation. This concept is well illustrated in the Seattle Proportional Risk Model [23]. A 2026 competing‐risk analysis further shows that age interacts with low LVEF to elevate VT/VF incidence alongside nonarrhythmic mortality [24]. However, over 40% of primary prevention ICDs in the United States are implanted in patients aged ≥ 70 years, indicating the necessity for careful consideration of age in conjunction with objective clinical markers to avoid overtreatment [25, 26].

#### 2.4.3. Prehospital Cardiac Arrest

Data from the MITRA plus registry, which analyzed 7111 STEMI patients with LVEF > 30% who survived hospital discharge, revealed that patients with prehospital cardiac arrest had a higher total mortality rate (13.6% vs. 8.7%) over a mean follow‐up of 13 months [27]. Another study further established that prehospital SCA is associated with significantly higher mortality rates compared to patients without such events [28]. Incorporating a history of prehospital arrest into risk models may help identify patients requiring closer monitoring and prompt ICD consideration.

### 2.5. Role of Noninvasive Modalities in Risk Stratification

#### 2.5.1. Imaging Modalities

Echocardiography remains the preferred modality for routine evaluation of structural SCD substrates, including left ventricular hypertrophy (LVH), which elevates arrhythmic risk through interstitial fibrosis disrupting conduction [29]. Global longitudinal strain (GLS) effectively predicts arrhythmic events across LVEF spectra, including preserved function [30]. Also, mechanical dispersion (MD), a novel measure of contractile dyssynchrony, independently forecasts such events regardless of LVEF [31]. These parameters offer cost‐effectiveness and broad availability, though they are constrained by interoperator variability and suboptimal fibrosis detection.

Speckle‐tracking echocardiography has been employed to assess myocardial tissue heterogeneity by measuring regional myocardial deformation. One study evaluated 79 patients with a first STEMI and LVEF ≤ 35% at three months, comparing strain values in the infarct core, border zone, and remote zone [32]. Over a median follow‐up of 46 months, 17% of patients reached a composite endpoint of appropriate ICD therapy or cardiac mortality. Although baseline strain values were similar, patients with adverse outcomes showed significantly impaired longitudinal strain at the border zone at 3 months [32].

Cardiac magnetic resonance (CMR) imaging with late gadolinium enhancement (LGE) has emerged as a robust tool for myocardial tissue characterization, with established prognostic value for cardiovascular events including SCD [33]. In an international cohort of ∼600 patients, Chan et al. demonstrated that greater LGE extent independently heightened SCD risk, with an odds ratio of 1.25 per 5% LGE increment [34].

Single‐photon emission computed tomography (SPECT) routinely assesses myocardial perfusion imaging (MPI) to evaluate coronary patency; among CAD patients, SPECT‐derived composite scores of fixed/reversible defects predict SCD independently, even in those with EF > 35% [35].

Positron emission tomography (PET), another MPI modality, quantifies scar, ischemia, or hibernating myocardium; in EF < 35% patients, PET‐identified scar, but not reversible ischemia, strongly correlated with ICD therapies and SCD [36].

#### 2.5.2. Cardiac Biomarkers

Biomarkers play a key role in refining SCD and arrhythmic risk prediction post‐MI by capturing inflammation, fibrosis, myocardial stress, and injury. Highly sensitive CRP (hs‐CRP), soluble ST2 (sST2), B‐type natriuretic peptide (BNP), and highly sensitive troponinT (hsTnT) independently predict SCD in CAD patients with preserved LVEF [37]. A recent study identified an SCD‐warning 3‐protein panel (coronin‐1A, haptoglobin, and complement factor D [CFD]) via proteomics in post‐MI cohorts [38]. This ELISA‐based model achieved C statistic = 0.752 for out‐of‐hospital SCD, surpassing LVEF ≤ 35% (C statistic = 0.548) and improving net reclassification, especially in LVEF‐preserved cases [38]. In a cohort of patients with prior MI and LVEF ≤ 30% undergoing ICD implantation, elevated preimplantation N‐terminal pro‐brain natriuretic peptide (NT‐proBNP) levels (> 2536 pg/mL) were independently associated with a higher likelihood of appropriate ICD therapies for VAs during one year of follow‐up (RR 7.7; *p* = 0.024), even after adjustment for LVEF, NYHA functional class, and other clinical covariates [39].

The mechanistic relationship between natriuretic peptides and arrhythmic risk remains incompletely understood, as their association with SCD may reflect underlying hemodynamic stress or ventricular remodeling rather than direct arrhythmogenic pathways, and prospective studies are needed to validate their role in post–MI risk stratification [40].

#### 2.5.3. Electrocardiographic (ECG) Predictors

Multiple noninvasive ECG markers, including heart rate (HR), QRS duration, ectopic ventricular activity, late potentials (LPs), repolarization abnormalities, and measures of autonomic function, have been investigated as potential tools to identify high‐risk, asymptomatic post–MI patients with preserved left ventricular systolic function [40]. These parameters may reflect underlying arrhythmogenic substrates and distinct patterns of arrhythmic vulnerability [41]. A prospective multicentre study validated a combined noninvasive ECG risk factors (NIRFs) plus programmed ventricular stimulation (PVS) for arrhythmic risk in post‐MI patients with LVEF > 40% [42]. NIRFs included premature ventricular complexes, NSVT, LPs, prolonged QTc, T‐wave alternans, reduced HR variability (HRV), and abnormal deceleration capacity/turbulence. NIRF‐guided PVS achieved 100% negative predictive value (NPV) and sensitivity for arrhythmic events, with high specificity and only 1.57% needing ICDs [42]. Another study involving 108 ICD patients (for both primary and secondary prevention) found that low HRV (≤ 6.5%), large QRS dispersion (> 39 ms) in patients with bundle branch block, and reduced T‐wave amplitude (< 0.4 mV) in patients without bundle branch block significantly increased the likelihood of appropriate ICD therapy [32]. Also, in 9511 middle‐aged adults free of cardiac disease, abnormal ECGs (16.3%) predicted 10‐year SCD (HR 1.62) and 30‐year risk (up to 6.6‐fold with multiples), identifying QRS ≥ 110 ms, QRST‐angle > 100°, LVH, T‐wave inversions, and early repolarization (ER) as key markers independent of age or comorbidities [43].

These findings confirm the role of ECG parameters as important, though not definitive, markers for arrhythmic risk.

### 2.6. Role of Invasive Modalities in Risk Prediction

#### 2.6.1. EPS

Early EPS provide a direct evaluation of the arrhythmogenic substrate by assessing the inducibility of VAs. In one study involving 360 patients undergoing EP testing nine days post‐MI, patients were categorized as EP‐positive (if VT with a cycle length ≤ 200 ms was induced) or EP‐negative. ICD implantation was recommended for EP‐positive patients (with 71% receiving implants), and after 2 years, the primary endpoint (sudden death or spontaneous VA) occurred in 22% of EP‐positive versus 4.3% of EP‐negative patients [11]. Another prospective study involving patients with LVEF ≤ 40% who underwent EPS between days 3–5 post‐MI demonstrated that a shorter coupling interval of the final extra stimulus (especially < 200 ms) was associated with a higher risk of spontaneous VAs. In addition, VT with right bundle branch block morphology was linked to increased all‐cause mortality [44]. A systematic review and meta‐analysis of EPS in post‐MI patients with reduced EF (< 40%) reported that inducibility of sustained VAs at EPS strongly predicts subsequent arrhythmic events, especially when sustained monomorphic VT inducibility was tested [45].

The routine use of EPS for post‐MI risk stratification is limited by its invasive nature, procedural risks, and the need for specialized equipment [40]. Importantly, a negative EPS does not eliminate the future risk of VAs. Consequently, EPS may be most valuable when used alongside noninvasive assessments to refine sudden death risk [46].

### 2.7. Role of Artificial Intelligence (AI) in Risk Stratification

Recent advancements have explored the utility of AI and machine learning algorithms in refining risk prediction in STEMI patients. One study employed an AI‐enabled ECG algorithm to predict left ventricular dysfunction postprimary PCI [47]. Serial ECGs from 637 patients, obtained at pre‐PCI, post‐PCI, and discharge, were analyzed to derive an AI‐based probability index. This index independently predicted LV dysfunction and was associated with increased rates of cardiac death and heart failure hospitalization [47]. Another study applied machine learning to arterial pressure (AP) wave data from 605 STEMI patients during primary PCI, achieving an area under the curve (AUC) > 0.80 for 1‐year mortality prediction when combined with demographic and clinical data [48]. Furthermore, a study developed machine learning models to predict inappropriate ICD therapy in 182 patients, identifying six key predictors (prior diagnosis of atrial arrhythmia, ischemic cardiomyopathy [ICM], absence of diabetes mellitus [DM], absence of cardiac resynchronization therapy [CRT], ST‐segment level at the J point in lead V3 (V3 STJ), and R‐wave amplitudes in lead V5 (V5R amp)) [49]. A 2025 meta‐analysis confirmed AI‐ECG’s high diagnostic performance for SCD (pooled sensitivity and specificity: 87%–99%, AUC 0.93–0.99 across methods), surpassing LVEF metrics and supporting NIRF enhancements, though prospective trials needed for post‐MI validation [50].

Wearable AI models excel at arrhythmia detection, including AF relevant to SCD risk. In a systematic review and meta‐analysis, 26 AF detection studies yielded pooled sensitivity 94.8% and specificity 96.96%, with deep neural networks superior performance (AUC = 0.981) over conventional machine learning algorithms (AUC = 0.961) [51].

AI applications in cardiac implantable electronic devices (CIEDs) like ICDs enhance arrhythmia detection, remote monitoring, and therapy optimization. Key applications encompass AI algorithms that precisely identify genuine arrhythmias while suppressing false positives from pacemakers and implantable monitors, neural network‐based models that forecast VAs weeks prior to ICD therapies, and individualized predictive models that determine heart failure patients likely to benefit from cardiac resynchronization therapy [52].

### 2.8. Integrative Risk Stratification and Future Directions

A multimodal risk stratification strategy that integrates patient‐specific factors with noninvasive and invasive assessments holds significant promise for improving ICD implantation decisions. Such an approach can effectively distinguish between patients with reversible myocardial damage, who may not benefit from ICD implantation, and high‐risk patients with preserved LVEF who are currently underrepresented in prophylactic ICD therapy, despite accounting for a substantial proportion of SCD cases [2]. While LVEF is likely to remain a crucial component of risk assessment [2, 40], it is essential to consider the dynamic nature of SCD risk, which evolves over time with changes in ventricular remodeling, heart failure progression, ischemic episodes, and alterations in the electrophysiological substrate [2, 53, 54]. Future research should aim to validate integrated predictive models in large‐scale, prospective studies while exploring the potential of AI to further enhance predictive accuracy.

While advanced imaging and electrophysiological testing may improve arrhythmic risk discrimination, factors such as cost, availability, operator dependency, and interpretability must be considered [55]. ECG LVEF remains widely accessible and inexpensive, whereas CMR and invasive EPS require greater resources. Therefore, future models should balance predictive performance with clinical feasibility.

## 3. Conclusion

While LVEF remains a cornerstone in post‐STEMI risk assessment, its limitations highlight the need for a broader, multimodal approach. By incorporating patient‐specific factors, noninvasive modalities, and judicious invasive assessments, clinicians can achieve more precise risk stratification. This comprehensive framework is essential for tailoring ICD therapy to those most likely to benefit, ultimately improving patient outcomes and optimizing resource utilization.

**Nomenclature**
STEMI: ST‐segment elevation myocardial infarction
SCD: Sudden cardiac death
MI: Myocardial infarction
ICD: Implantable cardioverter‐defibrillator
PCI: Percutaneous coronary intervention
LVEF: Left ventricular ejection fraction
Vas: Ventricular arrhythmias
CABG: Coronary artery bypass grafting
VT: Ventricular tachycardia
VF: Ventricular fibrillation
EF: Ejection fraction
HR: Hazard ratio
LV: Left ventricular
NYHA: New York Heart Association
SCA: Sudden cardiac arrest
NSVT: Nonsustained ventricular arrhythmias
EPS: Electrophysiology studies
U.S.: United States
LVH: Left ventricular hypertrophy
GLS: Global longitudinal strain
MD: Mechanical dispersion
CMR: Cardiac magnetic resonance
LGE: Late gadolinium enhancement
SPECT: Single‐photon emission computed tomography
CAD: Coronary artery disease
PET: Positron emission tomography
hs‐CRP: Highly sensitive CRP
sST2: Soluble ST2
BNP: B‐type natriuretic peptide
hsTnT: Highly sensitive troponinT
CFD: Complement factor D
NT‐proBNP: N‐terminal pro‐brain natriuretic peptide
ECG: Electrocardiographic
HR: Heart rate
LPs: Late potentials
NIRFs: Noninvasive risk factors
PVS: Programmed ventricular stimulation
NPV: Negative predictive value
HRV: Heart rate variability
ER: Early repolarization
EP: Electrophysiology
AI: Artificial intelligence
AP: Arterial pressure
AUC: Area under the curve
ICM: Ischemic cardiomyopathy
DM: Diabetes mellitus
CRT: Cardiac resynchronization therapy

## Author Contributions

Conceptualization and supervision: Masoumeh Sadeghi and Hamidreza Roohafza.

Data curation: Yasaman Shojaei and Masoumeh Sadeghi.

Investigation and project administration: Masoumeh Sadeghi.

Resources and software: Nasim Kakavand.

Validation: Sina Rouhani and Razieh Hassannejad.

Writing–original draft: Nasim Kakavand, Sina Rouhani, and Razieh Hassannejad.

Writing–review and editing: Masoumeh Sadeghi., Hamidreza Roohafza, Yasaman Shojaei, and Nasim Kakavand.

## Funding

The research reported in this publication was supported by the Elite Researcher Grant Committee under award number 996154 from the National Institute for Medical Research Development (NIMAD), Tehran, Iran.

## Disclosure

All authors have read and agreed to the published version of the manuscript.

## Ethics Statement

The project received ethical approval from the Ethics Committee of NIMAD under the reference number “IR.NIMAD.REC.1399.252.”

## Conflicts of Interest

The authors declare no conflicts of interest.

## Data Availability

Data sharing is not applicable to this article as no datasets were generated or analyzed during the current study.
